# Late reinfection with a different severe acute respiratory syndrome coronavirus-2 clade in a patient with refractory arterial hypertension: a case report

**DOI:** 10.1186/s13256-021-03071-2

**Published:** 2021-09-06

**Authors:** Javier García-Abellán, Antonio Galiana, Marta Fernández-González, Nieves Gonzalo-Jiménez, Montserrat Ruiz-García, Angela Botella, Joan Sanchis, Paula Mascarell, Selene Falcón, Mar Masiá, Félix Gutiérrez

**Affiliations:** 1 Infectious Diseases Unit, Hospital General de Elche and Universidad Miguel Hernández, Camino de la Almazara 11, 03203 Elche, Alicante Spain; 2 Microbiology Service, Hospital General de Elche, Camino de la Almazara 11, 03203 Elche, Alicante Spain

**Keywords:** SARS-CoV-2, Reinfection, COVID-19, Antibody response, Case report

## Abstract

**Background:**

Differentiating between persistent infection with intermittent viral shedding and reinfection with severe acute respiratory syndrome coronavirus 2 remains challenging. Although a small number of cases with genomic evidence of second infection have been reported, limited information exists on frequency and determinants of reinfection, time between infections, and duration of immunity after the primary infection.

**Case presentation:**

We report a reinfection with severe acute respiratory syndrome coronavirus 2 in a 52-year-old caucasian male whose primary infection was diagnosed in May 2020, during the first wave of the pandemic in Spain, and the second occurred 8 months later, in January 2021. We present a complete dataset including results from real-time polymerase chain reaction, serology, and genome sequencing confirming reinfection with a different clade. Noteworthy was that the patient was immunocompetent but had multiple cardiometabolic comorbidities, including refractory arterial hypertension, that might increase the individual risk in coronavirus disease 2019.

**Conclusions:**

This case of reinfection with severe acute respiratory syndrome coronavirus 2 occurring several months after the primary infection reports the longest time interval between reinfection and initial infection described to date. It raises concerns on the duration of protective immunity, suggesting that it may begin to wane in patients who acquired the initial infection during the first wave of the pandemic. The potential contributing role of arterial hypertension and cardiometabolic comorbidities as risk factors for reinfection deserves investigation.

## Background

A year after the coronavirus disease 2019 (COVID-19) pandemic began, uncertainty remains on the strength and duration of immunity after first severe acute respiratory syndrome coronavirus 2 (SARS-CoV-2) infection. Although only a small number of cases with genomic evidence of second infection have been reported thus far [1–7], recent data suggest that protective immunity may indeed be short-lived [8], thus raising concerns about the risk of reinfection as the pandemic progresses and immunity begins to wane.

Unfortunately, diagnosing reinfections remains challenging, requiring banking samples from first infection and performing genomic sequencing to distinguish reinfection from persistent viral shedding. As a result, limited data exist on frequency and determinants of SARS-CoV-2 reinfection. Therefore, studying every SARS-CoV-2 reinfection is important to describe the natural history of COVID-19 and to gain insight into the factors that may increase susceptibility to secondary infections.

Most reinfections described in the literature have occurred from 3 to 5 months after the initial episode, and most patients had antibodies against SARS-CoV-2 at the time of reinfection [7]. We have recently confirmed a second infection with a different clade of SARS-CoV-2 in a patient with multiple cardiometabolic risk factors for COVID-19 whose initial infection occurred 8 months before, in the first wave of the pandemic in Spain. The patient did not have anti-SARS-CoV-2 antibodies before onset of reinfection.

## Case presentation

A 52-year-old caucasian male with severe arterial hypertension was admitted to hospital on 10 May 2020, due to uncontrolled blood pressure with acute kidney failure. The patient did not have fever or respiratory symptoms. He had a past medical history of refractory hypertension with hypertensive heart disease and chronic kidney disease, morbid obesity, dyslipidemia and sleep apnea–hypopnea syndrome. He was receiving therapy with five antihypertensive agents of different classes, including angiotensin receptor blockers. Real-time polymerase chain reaction (PCR) screening for SARS-CoV-2 performed on a nasopharyngeal swab (NPS) before hospital admission was positive [cycle threshold value (Ct) = 30]. Chest radiograph did not show any abnormal findings, and all the laboratory biomarkers, including neutrophil-to-lymphocyte ratio, serum levels of C-reactive protein, interleukin 6, d-dimer, and ferritin, were within the normal range. After controlling blood pressure, he had an uneventful recovery and was discharged home 5 days after hospital admission. The patient did not receive corticosteroids, other immunomodulators, or antivirals.

He was contacted by phone 1 month after discharge and offered follow-up at the outpatient clinic to repeat serologic tests and NPS for SARS-CoV-2 at 2 and 6 months after hospital admission. Unfortunately, he missed the 2-month appointment but came to the clinic for the 6-month visit, on 14 December 2020. At that date, PCR on NPS was negative, and specific immunoglobulin G (IgG) against the SARS-CoV-2 internal nucleocapsid protein (N-IgG) and surface S1 domain of the spike protein (S-IgG) (anti-SARS-CoV-2 IgG ELISA, Euroimmun, Lubeck, Germany) were not present.

**Fig. 1:**
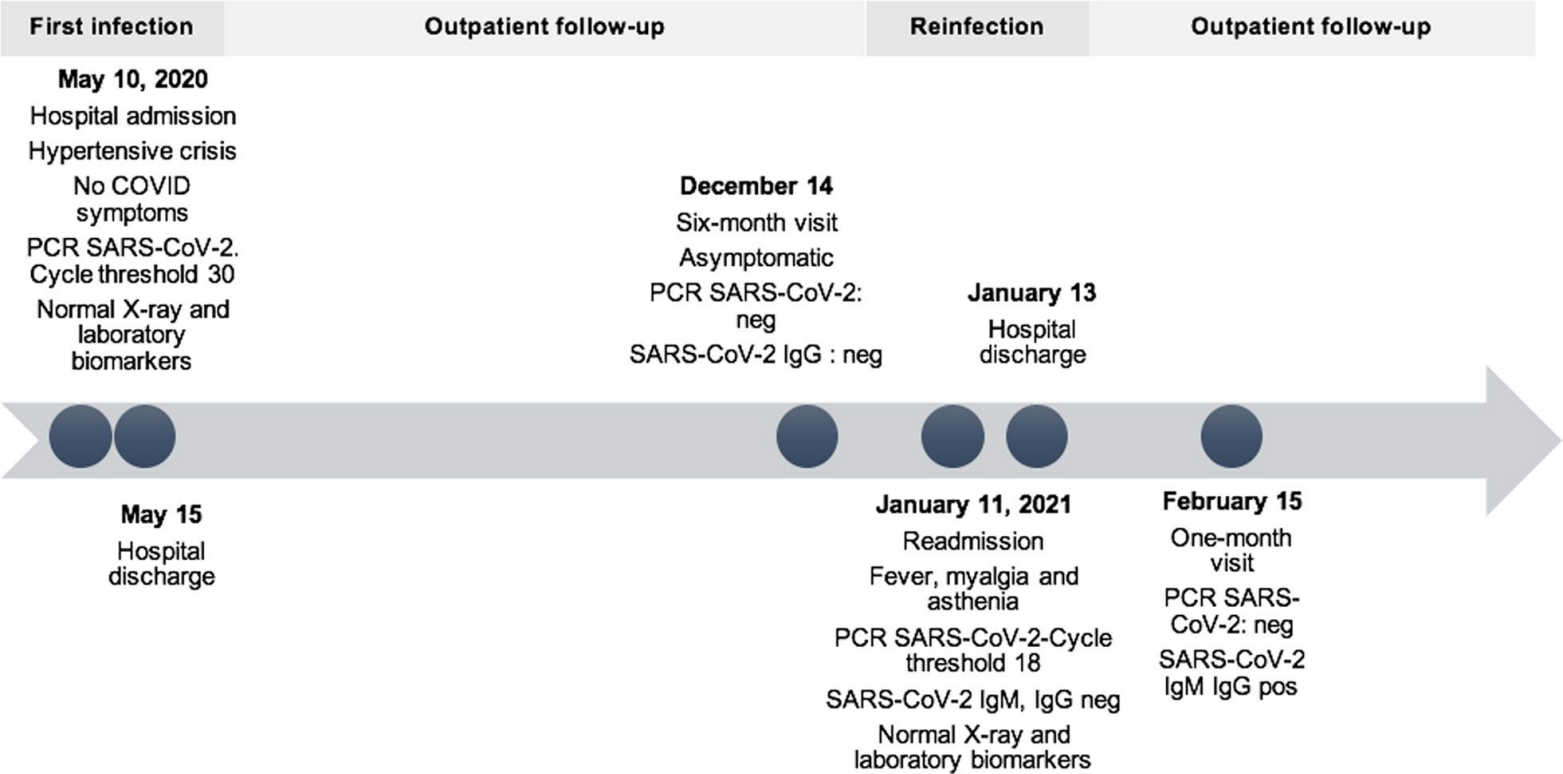
Timeline of events and clinical course. *PCR* real-time polymerase chain reaction, *IgG* immunoglobulin G, *IgM* immunoglobulin M

On 11 January 2021, 240 days after the first infection, he presented to hospital with a 3-day history of fever, myalgia, and asthenia. The patient had not received any vaccination against SARS-CoV-2. Chest radiograph was normal, and oxygen saturation was 95%. SARS-CoV-2 infection was confirmed by PCR testing on NPS with a Ct value of 18. He had a serum creatinine level of 1.8 mg/dL (normal value, 0.66–1.25 mg/dL) and a mild elevation of liver transaminases. The rest of the laboratory results were normal as with the initial infection. He did not have SARS-CoV-2-specific IgG antibodies (anti-SARS-CoV-2 S-IgG and N-IgG, Virclia Vircell, Granada, Spain and anti-SARS-CoV-2 S1/S2-IgG, Liaison DiaSorin, Saluggia, Italy). Specific IgM (anti-SARS-CoV-2 IgM, Liaison DiaSorin) and IgM + IgA (Virclia Vircell) were also negative. An extensive immunological investigation, including of lymphocyte subpopulations in peripheral blood, ruled out immunodeficiency. He received symptomatic care and oral dexamethasone with resolution of fever and myalgia, and was discharged on 13 January 2021. On 15 February 2021, PCR on NPS was negative. At that time, specific S-IgG, N-IgG and IgM + IgA (Virclia Vircell), and S1/S2-IgG (Liaison DiaSorin) were present. A summary of the clinical course observations made during first infection and reinfection is shown in Fig. 1.

## Genome sequencing

Genome sequencing of the viral genome was performed on stored aliquots of the NPS samples collected on 10 May 2020 (sample A) and 11 January 2021 (sample B) following the ARTIC SARS-CoV-2 amplicon sequencing protocol for MinION version V3 (https://www.protocols.io/view/ncov-2019-sequencing-protocol-bbmuik6w). The sequencing protocol uses 400 bp amplicons in a tiled fashion across the whole SARS-CoV-2 genome. After sample library synthesis, the samples were sequenced with the MinION nanopore sequencer (Oxford Nanopore Technologies, Oxford, UK) using the Ligation Sequencing Kit with the native barcoding expansion. The sequencing runs were performed using the flow cell FLO-MN106D and extended until both samples got 50,000 high-quality reads. Sequencing base calling was made using the nanopore guppy basecaller.

Downstream analysis was performed following the ARTIC nCoV-2019 bioinformatics protocol (https://artic.network/ncov-2019/ncov2019-bioinformatics-sop.html), a protocol that takes the output from the sequencing protocol to get the consensus genome SARS-CoV-2 sequences. The protocol follows the base calling step and makes demultiplexing sample sequences followed by sampling mapping step polishing and sequence consensus generation.

Phylogenetic analysis of both specimens was done using web server Nextstrain (https://nextstrain.org/), with the SARS-CoV-2 database Nextclade (https://clades.nextstrain.org/) to identify the clade, mutation calling, and phylogenetic placement of the SARS-CoV-2 genomes.

**Fig. 2:**
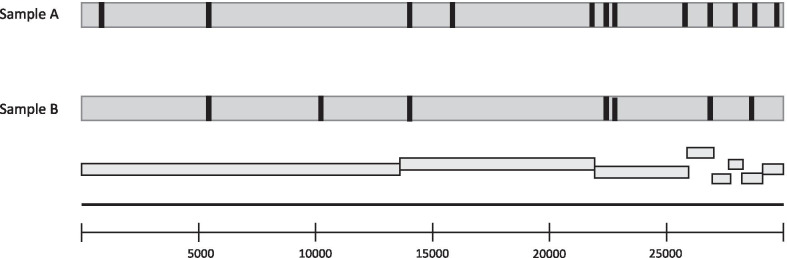
Single-nucleotide variants in the two NPS samples mapped on the SARS-CoV-2 genome. Sample A was obtained on 10 May 2020 and sample B on 11 January 2021

Single-nucleotide variants (SNVs) analysis against GISAID reference genomes showed differences between the two samples. Sample A was a member of the clade 20A EU1, as shown by its hallmark SNVs T445C, A23403G, and C28932T. Sample B was a member of the clade 20B, as its hallmark SNVs were T10836C, A23403G, and G28883C. Both samples shared some SNVs: G6167C, C14408T, A23122T, and A268016G. SNVs are presented in Table 1 and mapped on the SARS-CoV-2 genome in Fig. 2. Phylogenetic analysis of both samples against GISAID genomes database using Nextclade web server analysis is shown in Fig. 2.

**Table 1:** Single-nucleotide variants analysis versus reference genome, and amino-acid substitutions in the two samples

Nucleotide mutations versus reference genome	Sample*		Amino-acid substitutions
	A	B	
T445C	Yes	No	–
G6167C	Yes	Yes	ORF1a: V1968L
T10836C	No	Yes	ORF1a: V3524A
C14408T	Yes	Yes	ORF1b: P314L
T16213C	Yes	No	ORF1b: Y916H
T22669C	Yes	No	–
A23122T	Yes	Yes	–
A23403G	Yes	Yes	S: D614G
A25900G	Yes	No	ORF3a: T170A
A268016G	Yes	Yes	–
C27944T	Yes	No	–
G28883C	No	Yes	N: G204R
C28932T	Yes	No	N: A220V
G29645T	Yes	No	–

^*^Sample A was obtained on 10 May 2020 and sample B on 11 January 2021

## Discussion and conclusions

This case presentation adds to the still limited knowledge on second infections with SARS-CoV-2. Here, a complete dataset including results from PCR, serology, and genome sequencing is provided, allowing us to confirm reinfection with a different clade 8 months after an asymptomatic initial infection. The first infection was with the 20A clade, and the reinfection with the 20B. The two clades are characterized by a D614G substitution in the S gene, and reflect the most frequent circulating strains in Spain at that time [9]. The underlying cardiometabolic comorbidities in this case, in particular the presence of severe refractory hypertension, give rise to doubts to whether this condition, one of the most common pre-existing comorbidities in patients with COVID-19 [10], could eventually increase the individual risk of SARS-CoV-2 reinfection.

The interval between reinfection and initial infection was the longest reported so far in the literature and probably reflects waning immunity over time. Recent studies have emphasized rapid decay of anti-SARS-CoV-2 antibodies with large portions of a study population sero-reverting within a few months [8]. Indeed, in this case report, we documented that anti-SARS-CoV-2 antibodies were not present shortly before onset of the second infection and at the time of presentation, thus highlighting the role of humoral immunity in the protection against reinfection.

As in other published cases [11], in the patient presented herein, the clinical presentation was more intense and severe during the second episode than the first infection, requiring treatment with dexamethasone and leading to the production of SARS-CoV-2-specific IgM and IgG antibodies.

This case of reinfection in an immunocompetent host several months after an asymptomatic infection raises concerns on the duration of protective immunity and suggests that immunity may begin to wane in patients who acquired the initial infection during the first wave of the pandemic. The potential contributing role of arterial hypertension and cardiometabolic comorbidities as risk factors for reinfection deserves investigation.

## Data Availability

Genome sequencing results are available from the corresponding author on reasonable request.

## Abbreviations

SARS-CoV-2: Severe acute respiratory syndrome coronavirus 2
COVID-19: Coronavirus disease 19
PCR: Polymerase chain reaction
NPS: Nasopharyngeal swab
IgG: Immunoglobulin G
SNV: Single nucleotide variant
